# Safety and efficacy of ipilimumab to treat advanced melanoma in the setting of liver transplantation

**DOI:** 10.1186/s40425-015-0066-0

**Published:** 2015-06-16

**Authors:** Rita E Morales, Alexander N Shoushtari, Michelle M Walsh, Priya Grewal, Evan J Lipson, Richard D Carvajal

**Affiliations:** Columbia University College of Physicians and Surgeons, 630 W. 168th Street, New York, NY 10032 USA; Melanoma and Immunotherapeutics Oncology Service, Memorial Sloan Kettering Cancer Center, 300 East 66th Street, New York, 10065 NY USA; The Herbert Irving Comprehensive Cancer Center, Columbia University Medical Center, 177 Fort Washington Avenue, Suite 6-435 Garden North, New York, NY 10032 USA; Division of Liver Diseases, Mount Sinai School of Medicine, One Gustave L. Levy Place, Box 1104, New York, NY 10029 USA; Johns Hopkins University School of Medicine, Sidney Kimmel Comprehensive Cancer Center, 400 N. Broadway, Baltimore, MD 21231 USA

## Abstract

Ipilimumab is a first-in-class immunological checkpoint blockade agent and monoclonal antibody against Cytotoxic T-Lymphocyte Antigen 4 (CTLA-4) that has demonstrated survival benefit and durable responses in patients with metastatic melanoma. To date, solid organ transplant recipients have been excluded from clinical trials with cancer immunotherapies on the basis of their concurrent treatment with immunosuppressive agents. We present the first case to our knowledge of a patient with advanced cutaneous melanoma receiving ipilimumab status post orthotopic liver transplantation with a partial response. Transaminitis was observed 4 months after administration of ipilimumab that resolved with close observation. No evidence of graft rejection has been observed to date. This case advocates for further investigation of the safety and efficacy of cancer immunotherapies in solid organ transplant recipients.

## Background

Patients presenting with melanoma after solid organ transplantation present unique challenges to oncologists. There are several important considerations in this setting, including the risk of injury to the allograft with the administration of anticancer therapy and the scarcity of published data regarding the clinical management of advanced melanoma in organ transplant recipients. This is especially relevant given the recent development of immunomodulatory therapies for the treatment of metastatic melanoma. Ipilimumab is a first-in-class immunological checkpoint blockade agent approved by the United States Food and Drug Administration (FDA) in 2011 for the treatment of unresectable or metastatic melanoma. Two phase III studies have demonstrated improved overall survival rates with ipilimumab as compared to dacarbazine or a peptide vaccine control [1,2], Long-term disease control has been documented in approximately 20% of individuals treated [3]. Given the success of ipilimumab in advanced melanoma, it is important to consider the safety and efficacy of this agent in solid organ transplant recipients. The only published experience administering drug to a solid organ transplant recipient is by Lipson and colleagues [4], who reported two cases of successful administration of ipilimumab to patients with kidney transplants. Here, we present the first case describing the use of ipilimumab in a patient with a liver transplant.

## Case presentation

A 67-year-old man with a history of hepatitis C virus (HCV) and hepatocellular carcinoma (HCC) underwent orthotopic liver transplantation in 2006. He presented with decompensated liver disease marked by ascites and hepato-renal syndrome leading to renal failure. He was on hemodialysis prior to transplant and had a Model for End-Stage Liver Disease (MELD) score of 35 at time of transplant. His donor was a 49-year-old man with no history of malignancy. Explant pathology revealed established cirrhosis with a 2.5 cm moderately differentiated HCC with evidence of microvascular invasion. After transplantation, he maintained stable liver function on an immunosuppressive regimen of tacrolimus and mycophenolate mofetil. He underwent two post-transplant liver biopsies, which revealed HCV recurrence: Grade 1, Stage 1 in 2008 and Grade 1, Stage 2 in 2010. His liver enzymes remained in the normal range at this time. He underwent HCC surveillance at 6 monthly intervals for the first 2 years after transplant and yearly thereafter for the next 3 years.

In 2007, a small pigmented skin lesion above his left eyebrow grew larger and became ulcerated. A biopsy in September 2009 revealed an ulcerated melanoma at least 0.7 mm in Breslow thickness. Wide local excision with sentinel lymph node biopsy performed in November 2009 demonstrated residual ulcerated melanoma, 2.51 mm thick, Clark Level IV, with a mitotic index of 11/mm2 and no lymphovascular or perineural invasion. Two sentinel nodes were negative for disease. Initial clinical staging by American Joint Committee on Cancer (AJCC) 7^th^ edition was T3bN0, Stage IIB. In 2010, HCC surveillance imaging revealed a 5 cm right adrenal mass that was subsequently resected. The pathology revealed metastatic HCC. At this point his immunosuppression was switched from tacrolimus to rapamycin 3 mg daily and he was continued on mycophenolate mofetil 500 mg twice daily.

The patient underwent active surveillance with clinical exams and imaging studies until October 2013, when he noticed a swollen mass in the left parotid region. A needle biopsy revealed melanoma wild-type for *BRAF* and c*KIT*. A positron emission tomography/computerized tomography (PET/CT) scan performed in November 2013 demonstrated prominent focal hypermetabolic activity in bilateral lung nodules, bony foci and a parotid lesion, consistent with M1c disease. Magnetic resonance imaging (MRI) of the brain showed no evidence of metastases. A repeat scan in January 2014 showed increasing nodal and bony disease and new hepatic metastases in the allograft. At that point, he was initiated on therapy with paclitaxel. His rapamycin was reduced from 3 mg to 1 mg daily and mycophenolate mofetil was discontinued, given prior clinical reports documenting tumor regression with reduction of immunosuppression [5,6]. He completed 5 cycles of chemotherapy as well as 14 of 20 planned fractions of palliative radiotherapy to the hip before experiencing multifocal disease progression in April 2014, with increasing disease burden in the lungs, mediastinal lymph nodes, liver and spleen.

**Figure 1:**
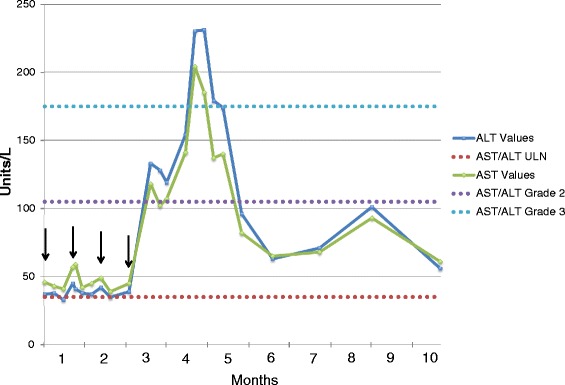
Demonstration of changes in AST/ALT values over time with administration of ipilimumab; AST and ALT levels were measured weekly during the administration of ipilimumab. Sixteen weeks following initial induction, AST and ALT levels peaked at Grade 3. Resolution to Grade 1 was seen at Week 20, and monthly monitoring of liver function tests has been performed since, with continued evidence of stability. Arrows denote the times of administration of ipilimumab.

After a multidisciplinary team-based discussion including both medical oncology and transplant medicine, the decision was made to begin therapy on ipilimumab while maintaining rapamycin at 1 mg daily. Given the increased risk of graft rejection, the treatment plan included weekly monitoring of liver function tests. The patient received his four induction doses of ipilimumab 3 mg/kg between April 2014 and July 2014. The patient experienced a mild non-pruritic rash on his torso after the second infusion, which lasted one week and resolved with topical steroids. The aspartate aminotransferase (AST), alanine aminotransferase (ALT) and alkaline phosphatase were mildly elevated at baseline (Common Terminology Criteria for Adverse Events [CTCAE] v4.0 Grade 1) and remained stable throughout the course of treatment. Total bilirubin was within normal limits at baseline and remained normal throughout the course of treatment. The fourth and final dose of ipilimumab was administered at week 10, on 7/1/2014. On week 12, a Grade 2 transaminitis and alkaline phosphatase elevation developed, with no associated hyperbilirubinemia. The patient was managed conservatively with frequent laboratory tests. AST and ALT levels peaked at a Grade 3 on week 16 with stabilization or improvement the following week without intervention (Figure 1). Because the patient remained asymptomatic and compliant with weekly laboratory testing, it was decided to maintain him on close monitoring and defer initiation of corticosteroids. Alkaline phosphatase levels peaked at week 17, but remained Grade 1 throughout the course of treatment. At last monitoring in February 2015, this value had returned to within normal limits. Total bilirubin has remained within normal limits since the induction of treatment. By week 20, all lab values resolved to Grade 1, and conservative management was continued. The patient declined a recommended liver biopsy. The patient remains stable 10 months following induction of treatment.

**Figure 2:**
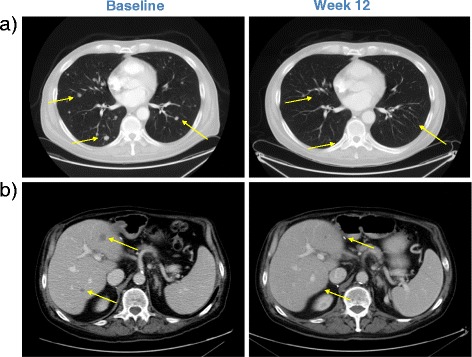
Demonstration of radiographic response to ipilimumab; CT scans performed prior to and 12 weeks after initiation of therapy demonstrate regression of lung and liver metastases (arrows).

Repeat CT scans conducted in July 2014, after his fourth infusion of ipilimumab, showed dramatic tumor regression in the lungs (Figure 2a) as well as the liver (Figure 2b). Clinically, the patient continues to feel well and is undergoing continued close monitoring for both his liver function and disease status.

## Conclusions

Compared to the general population, organ transplant recipients (OTRs) have an estimated 2.4 fold increased risk of developing melanoma [7]. Prognosis for these patients is also estimated to be worse than those with non-transplant associated melanomas. Brewer and colleagues [8] performed the largest retrospective review of patients with melanoma arising post-transplant to date, which demonstrated decreased overall survival for OTRs with localized melanomas with Breslow thickness >1.5 mm or Clark Levels III and IV, compared to immunocompetent controls. They also found that the degree of immunosuppression was negatively correlated with survival.

Given the increased risk for melanoma in solid organ transplant patients as well as poor outcomes, the development of treatment strategies for this population is necessary. Two previously published case reports have described complete responses of metastatic melanoma after withdrawal of immunosuppressive medications in aplastic anemia and myasthenia gravis, without significant adverse effects related to immune reconstitution [5,6]. However, withdrawal of immunosuppression may not be feasible in the setting of solid organ transplantation. Therefore, therapeutic options for the advanced melanoma must include consideration of agents such as ipilimumab.

There are at least two barriers to the use of immune checkpoint inhibitors such as ipilimumab in OTRs. Firstly, because ipilimumab ostensibly requires a competent T-cell population to carry out its antitumor function, the co-administration of immunosuppressive agents such as rapamycin to prevent graft rejection may impact the efficacy of ipilimumab in this population. In this case, the patient was maintained on low-dose immunosuppression with rapamycin throughout his course of treatment with ipilimumab. However, given his marked radiographic response to treatment, it appears that the efficacy of the ipilimumab was not significantly affected by his concurrent immunosuppression.

A second barrier to treatment in cases such as this stems from the ability of drugs such as ipilimumab to activate T cells that may be specific for non-self antigens expressed by the allograft, precipitating organ rejection. In our patient, there was initial concern for an adverse immune reaction to treatment, especially given the rise in liver function tests that was seen after 12 weeks of treatment. However, with no further ipilimumab administration and expectant observation, the patient’s transaminitis resolved to near baseline levels within 8 weeks.

Given these challenges, it is important to identify which patients may tolerate a reduction of immunosuppression as well as the use of immunomodulatory agents. Although no definitive prognostic markers have been identified, several studies have identified increased length of time since transplantation as a predictive factor for tolerance of immunosuppression withdrawal [9,10]. In addition, compared with other organs such as the heart and lung, liver grafts are considered to be the least immunogenic organs for transplant and thus can sustain less aggressive immunosuppressive regimens [11]. The patient described above received his liver transplant 8 years before induction with ipilimumab, and was thus able to tolerate reductions to his immunosuppressive regimen before induction with ipilimumab. This, along with close monitoring of liver function and immunosuppressive drug levels, may have contributed to our success in treating him both safely and effectively with this drug.

Although further study in a large patient cohort is warranted, this case has demonstrated the feasibility and efficacy in administering ipilimumab to a liver transplant recipient, and may suggest that liver transplant recipients several years post-transplant may be appropriate candidates for trials with immunomodulatory treatments. Our small sample size underscores the importance of reporting safety and efficacy data for ipilimumab in the solid tumor organ transplant population. Furthermore, the promising data from trials of programmed cell death-1 (PD-1) and programmed death-ligand 1 (PD-L1) antibodies in metastatic melanoma make it especially important to study the potential effects of immune checkpoint inhibitors in immunocompromised patients. The risks surrounding graft rejection in the setting of immune activation with these agents must be strongly considered when making treatment decisions in this population.

## Consent

Written informed consent was obtained from the patient for publication of this case report and any accompanying images. A copy of the written consent is available for review by the Editor-in-Chief of this journal.

## Abbreviations

HCV: Hepatitis C Virus
HCC: Hepatocellular carcinoma
CTCAE: Common Terminology Criteria for Adverse Events
AST: Aspartate aminotransferase
ALT: Alanine aminotransferase
OTR: Organ transplant recipient

## References

[1] Hodi FS, O’Day SJ, McDermott DF, Weber RW, Sosman JA, Haanen JB (2010). Improved survival with ipilimumab in patients with metastatic melanoma. N Engl J Med.

[2] Robert C, Thomas L, Bondarenko I, O’Day S, Weber J, Garbe C (2011). Ipilimumab plus dacarbazine for previously untreated metastatic melanoma. N Engl J Med.

[3] Schadendorf D, Hodi FS, Robert C, Weber JS, Margolin K, Hamid O et al. Pooled Analysis of Long-Term Survival Data From Phase II and Phase III Trials of Ipilimumab in Unresectable or Metastatic Melanoma. J Clin Oncol 2015. doi:10.1200/JCO.2014.56.2736. 10.1200/JCO.2014.56.2736 PMC5089162 25667295

[4] Lipson EJ, Bodell MA, Kraus ES, Sharfman WH (2014). Successful administration of ipilimumab to two kidney transplantation patients with metastatic melanoma. J Clin Oncol.

[5] Dillon P, Thomas N, Sharpless N, Collichio F (2010). Regression of advanced melanoma upon withdrawal of immunosuppression: case series and literature review. Med Oncol.

[6] Hodi FS, Granter S, Antin J (2005). Withdrawal of immunosuppression contributing to the remission of malignant melanoma: a case report. Cancer Immun.

[7] Dahlke E, Murray CA, Kitchen J, Chan AW (2014). Systematic review of melanoma incidence and prognosis in solid organ transplant recipients. Transplant Res.

[8] Brewer JD, Christenson LJ, Weaver AL, Dapprich DC, Weenig RH, Lim KK (2011). Malignant melanoma in solid transplant recipients: collection of database cases and comparison with surveillance, epidemiology, and end results data for outcome analysis. Arch Dermatol.

[9] Benitez C, Londono MC, Miquel R, Manzia TM, Abraldes JG, Lozano JJ (2013). Prospective multicenter clinical trial of immunosuppressive drug withdrawal in stable adult liver transplant recipients. Hepatology.

[10] de la Garza RG, Sarobe P, Merino J, Lasarte JJ, D’Avola D, Belsue V (2013). Trial of complete weaning from immunosuppression for liver transplant recipients: factors predictive of tolerance. Liver Transpl.

[11] Sanchez-Fueyo A, Strom TB (2011). Immunologic basis of graft rejection and tolerance following transplantation of liver or other solid organs. Gastroenterology.

